# Neural mechanisms underlying peripheral facial nerve palsy: A protocol for systematic review and meta-analysis of neuroimaging studies

**DOI:** 10.1097/MD.0000000000032110

**Published:** 2022-12-02

**Authors:** Dong Hyuk Lee, Bo-In Kwon, Jun-Sang Yu, Sang Kyun Park, Joo-Hee Kim

**Affiliations:** a Department of Anatomy, College of Korean Medicine, Sangji University, Wonju-si, Gangwon-do, Republic of Korea; b Research institute of Korean medicine, Sangji University, Wonju-si, Gangwon-do, Republic of Korea; c Department of Pathology, College of Korean Medicine, Sangji University, Wonju-si, Gangwon-do, Republic of Korea; d Department of Sasang Constitutional Medicine, College of Korean Medicine, Sangji University, Wonju-si, Gangwon-do, Republic of Korea; e Department of Meridian & Acupoints, College of Korean Medicine, Sangji University, Wonju-si, Gangwon-do, Republic of Korea; f Department of Acupuncture and Moxibustion Medicine, College of Korean Medicine, Sangji University, Wonju-si, Gangwon-do, Republic of Korea.

**Keywords:** Bell’s palsy, functional magnetic resonance imaging, neuroimaging, peripheral facial nerve palsy, systematic review

## Abstract

Peripheral facial nerve palsy is a general term used to describe diseases that cause lower motor neuron lesions in the facial nerve. The most common cause of peripheral facial nerve palsy is idiopathic Bell’s palsy (BP), accounting for 60% to 70% of all peripheral facial nerve palsy cases. Idiopathic BP is found in various medical conditions, such as ischemia, viral infection, and neuroinflammation; however, the specific pathophysiology remains unclear. Neuroimaging techniques help understand the neural mechanisms underlying various neurological disorders. In recent years, several studies have applied functional neuroimaging to investigate alterations of brain function in BP patients. However, the results have been inconsistent due to differences in methodology and quality. Moreover, systematic reviews of studies on functional neuroimaging for BP are insufficient. Therefore, the present study aims to verify the neural mechanisms of brain alterations and draw conclusions about the functional reorganization in patients with BP.

**Methods:**

This review includes all suitable studies published on or before October 31, 2022. A thorough search will be conducted using the following databases: MEDLINE, Cochrane Library, Excerpta Medica Database (EMBASE), China Knowledge Resource Integrated Database (CNKI), Korean Medical database (KMBASE), Korean Studies Information Service System (KISS). Clinical studies of BP using functional neuroimaging will be selected. We will apply a coordinate-based meta-analysis because most individual neuroimaging studies provide their results as coordinates in the standard space. The primary outcomes will include the types of functional neuroimaging methods and alterations of brain function in BP patients. The secondary outcomes will include the information about clinical measurement of the disease. Study selection, data extraction, and risk of bias assessment will be conducted. If possible, heterogeneity tests, data synthesis, and subgroup analyses will be conducted.

**Results:**

The study will analyze the alterations in brain activity and worsening of clinical symptoms caused by idiopathic BP.

**Conclusion:**

The aim of this study is to investigate functional reorganization of brain alterations in patients with BP. This review will improve the understanding of the neural mechanisms of BP based on the most recent publications through extensive data retrieval. If sufficient data are collected, a sensitivity analysis is performed to verify the robustness of the conclusions.

## 1. Introduction

Peripheral facial nerve palsy is a generic term for diseases that cause lower motor neuron lesions in the facial nerve. It can result from various medical conditions such as infection, trauma, autoimmune disease, and neoplasm. The most common cause of peripheral facial nerve palsy is idiopathic Bell’s palsy (BP).^[1]^ BP accounts for 60% to 70% of all peripheral facial nerve palsy cases, with an estimated incidence of 20 to 30 cases per 100,000 people.^[2,3]^ Neuroinflammation, ischemia, and viral infections such as varicella-zoster virus and herpes simplex virus type 1 have been suggested as etiologies of BP, but the specific mechanisms remain unclear.^[4]^

Facial nerve palsy can be classified into acute (minutes to days), subacute (days to weeks), and chronic (longer than weeks) based on the time course of disease onset.^[5]^ Although the prognosis of BP is relatively good, 20% to 30% of BP patients reportedly suffer from incomplete recovery and sequelae, including facial synkinesis, contracture, and paresthesia.^[6,7]^ Electrodiagnostic tests, such as electromyography and electroneuronography, are used to determine the prognosis of facial nerve palsy by measuring the degree of nerve damage.^[8]^

The development of neuroimaging techniques has shed light on the neural mechanisms underlying various diseases.^[9–11]^ In particular, in the case of peripheral nerve disorders, such as BP, functional brain changes due to neuroplasticity can be induced even if the structural alterations are not clear.^[12–14]^ Therefore, to identify the pathophysiology of the disease, it is essential to use neuroimaging techniques that can explore functional changes in the brain. Functional neuroimaging methods include functional magnetic resonance imaging (fMRI), positron emission tomography, single-photon emission computed tomography, and magnetic and optical imaging.^[15]^ Among them, fMRI is the most widely employed modality in the research field because of its excellent spatiotemporal resolution and noninvasive properties.^[16–19]^

Notably, various clinical studies using functional imaging have been conducted to reveal the neural mechanisms underlying BP. However, the diversity of these studies’ quality and study design has led to inconsistent results. Moreover, systematic literature reviews of studies using functional neuroimaging for BP are insufficient. Therefore, this protocol has been designed to verify the neural mechanisms of BP in studies using functional brain imaging for a further systematic review. In this study, clinical trials applying functional neuroimaging in BP will be systematically evaluated to secure a basis for the neural mechanisms underlying peripheral facial nerve palsy.

## 2. Methods

This protocol is based on the Preferred Reporting Items for Systematic Reviews and Meta-analyses (PRISMA-P) statement guidelines. The PRISMA-P checklist of this protocol was sent at the submission stage. Our protocol has been registered on the PROSPERO registration (number: CRD42022299736). The results of this analysis will be published later in the journal. Ethical approval was not deemed necessary because this is a literature-based study.

### 2.1. Eligibility criteria

#### 2.1.1. Types of studies

This review will include clinical studies on BP using functional neuroimaging to reveal the characteristics or alterations of the disease. Case reports, animal studies, letters, editorials, and conferences will be excluded.

#### 2.1.2. Types of participants

Only patients with BP will be included in the review. The study will contain BP patients at all stages from the acute to the chronic stage. Patients with facial nerve palsy due to other causes will be excluded.

#### 2.1.3. Types of interventions

This review will cover functional neuroimaging techniques as interventions or modalities because we intend to focus on the functional reorganization or neuroplasticity that occurs during the disease course.

#### 2.1.4. Type of comparators

No limitations will be placed for the comparators. No treatment, treatment such as steroids, anti-viral drugs, acupuncture, physical therapy, or other treatments will be eligible.

#### 2.1.5. Types of outcomes

The types of functional neuroimaging techniques and neuroimaging results of indices, such as functional connectivity, regional homogeneity, and fractional amplitude of low-frequency fluctuation in fMRI, will be included. Second, the outcome will include clinical measurements of facial nerve palsy, such as the Stennert grading system or the House-Brackmann scale.

### 2.2. Search methods for identification of studies

#### 2.2.1. Information sources and search strategy

A systematic literature search will be conducted using the following databases: MEDLINE, Cochrane Library, Excerpta Medica Database (EMBASE), China Knowledge Resource Integrated Database (CNKI), Korean Medical database (KMBASE), Korean Studies Information Service System (KISS), ScienceON (database of Korea Institute of Science and Technology information), and Oriental Medicine Advanced Searching Integrated System (OASIS). All publications published on October 31, 2022, will be searched. There will be no restrictions on language or publication type during the search. The search terms to be used are listed as follows: “BP,” “peripheral facial nerve palsy,” “neuroimaging,” “fMRI,” “functional MRI,” “MRI,” “positron emission tomography,” “single-photon emission computed tomography,” “functional connectivity,” “blood oxygen level dependent,” “functional image,” “blood oxygen level dependent,” “resting state,” “regional homogeneity,” “Centrality,” “Independent Component Analysis,” and “amplitude of low-frequency fluctuation.” The detailed search strategies are presented in Table 1.

**Table 1:** Search strategy for MEDLINE.

Number	Search terms
1	Facial nerve palsy
2	Bell’s palsy
3	Peripheral facial nerve palsy
4	Facial palsy
5	Idiopathic facial palsy
6	1 or 2 or 3 or 4 or 5
7	Neuroimag*
8	Magnetic resonance imag*
9	MRI
10	PET
11	DTI
12	fMRI
13	Resting state
14	Functional imag*
15	Functional connectivity
16	SPECT
17	Blood oxygen level dependent
18	BOLD
19	Regional homogeneity
20	ReHo
21	Amplitude of low-frequency fluctuation
22	ALFF
23	fALFF
24	Voxel-based analys*
25	Centrality
26	ICA
27	Independent component analysis
28	ROI
29	Region of interest
30	OR #7-29
31	#6 AND #30

BOLD = blood oxygen level dependent, fMRI = functional magnetic resonance imaging, PET = positron emission tomography, SPECT = single-photon emission computed tomography.

### 2.3. Data collection and analysis

#### 2.3.1. Study selection

**Figure 1.**
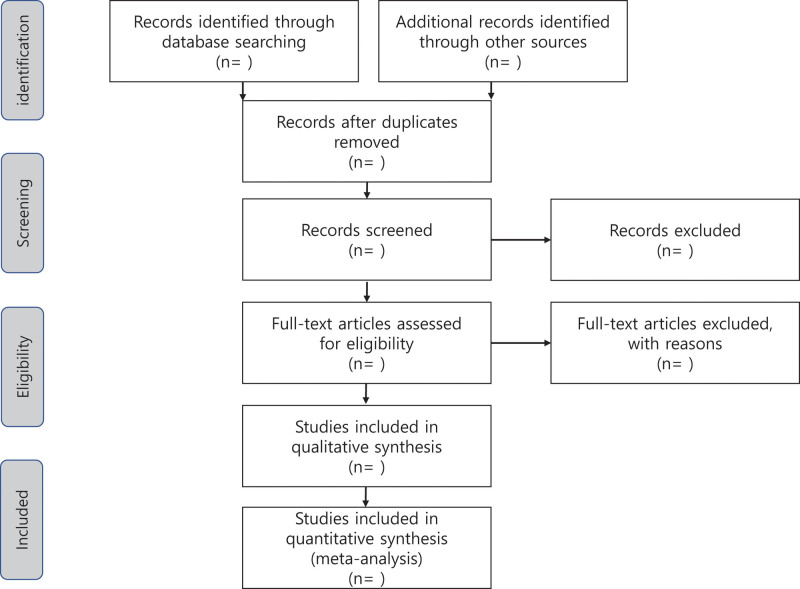
Flow diagram of the study selection.

First, 2 researchers will independently screen the search results and identify tentatively appropriate citations. Second, they will download the full texts of the articles and determine their eligibility. Finally, they will record the reasons for the exclusion of articles and resolve any disagreements via discussion. EndNote 20 software will be used to filter the articles. A flow diagram of the study selection process is shown in Figure 1.

#### 2.3.2. Data extraction and management

Two reviewers will independently review the included articles and extract the following information: general characteristics (title, first author, publication year, country, and journal name), subject characteristics (baseline data, age, sex, and BP stage), other properties (sample size and study design), clinical outcomes, and neuroimaging results. If the above data in an article are incomplete, we will contact the author to request the missing information.

#### 2.3.3. Addressing missing data

For missing data, we will try to contact corresponding authors for further information. Otherwise, we will proceed using existing information and conduct sensitivity analysis to address potential effect of missing data.

#### 2.3.4. Quality assessment

The quality of each study will be evaluated using a 12-point checklist, based on previous neuroimaging meta-analyses.^[20]^ The checklist contains 3 categories: participants (items 1–4), methods of imaging acquisition and analysis (items 5–10), and the results and conclusions (items 11–12). Each item is scored 1, 0.5, or 0, based on whether the criteria are fully, partially, or not met, respectively.

#### 2.3.5. Data synthesis and statistical analysis

We will apply a coordinate-based meta-analysis because most individual neuroimaging studies provide their results as coordinates in the standard space. Furthermore, coordinate-based meta-analysis entail various specific methods, such as activation likelihood estimation, kernel density analysis, Gaussian process regression, parametric voxel-based meta-analysis, and signed differential mapping.^[21,22]^ We will utilize GingerALE software version 3.0.2 (http: http://brainmap.org/ale/), which implements the latest ALE algorithm.^[23]^ ALE is an approach of meta-analysis that collects activation regions reported in neuroimaging studies and creates 3D Gaussian probability distributions for each focus to compute activation maps. Finally, the convergence of foci is assessed by testing against the null-hypothesis of random spatial maps. We will summarize Region of Interests with significant changes of brain activation in the included studies and significant neural substrates of clinical measurements. The peak coordinates in the Talairach space are transformed into the MNI space (or vice versa). The standard thresholds will be set as uncorrected *P* < .005 and voxel size ≥ 10 to balance the sensitivity and specificity.

#### 2.3.6. Meta-regression or subgroup analysis

Meta-linear regression analysis based on the selected meta-analysis method will be performed if necessary to explore the effect of age, sex, and disease duration in BP patients. The threshold of *P* < .005 will be set so that brain areas with a significant association in the alterations of brain function can be reported. When appropriate studies are included, a subgroup analysis will be conducted to explore the potential causes of heterogeneity.

#### 2.3.7. Publication bias

If sufficient research is included, publication bias will be evaluated through a funnel plot using the Egger test. Publication bias will be considered significant if the result of the Egger test reveals *P* < .05.

#### 2.3.8. Sensitivity analysis

When sufficient studies are available, we will perform a jackknife sensitivity analysis to test the repeatability of the results. In jackknife analysis, the statistical analysis is performed iteratively by discarding different datasets each time.

## 3. Discussion

Facial nerve palsy greatly affects patients’ quality of life. The facial appearance is very important for esthetics, so patients with facial nerve palsy experience great difficulties in their social life, as well as physical discomfort and psychological stress.^[24,25]^ Fortunately, BP, the main cause of peripheral facial nerve palsy, generally follows a benign course. However, approximately 30% of patients suffer from sequelae such as unrecovered palsy, facial spasm, synkinesis, and contracture of facial muscles.

The mechanism of the disease is considered to be associated with viruses, neuroinflammation, immune response, and ischemia; however, more in-depth research on disease mechanisms is still needed. Currently, neuroimaging techniques are increasingly utilized to unravel the pathophysiology and neural mechanisms of BP because of their noninvasive nature and excellent spatiotemporal resolution. Functional neuroimaging can be particularly useful for the study of diseases that do not structurally alter the brain, but lead to functional brain changes due to brain reorganization or neuroplasticity. In the fields of facial nerve palsy, steady research has assessed the neural mechanisms using fMRI. However, it is difficult to interpret the results in an integrated manner owing to the heterogeneous features of the study, such as differences in study design, quality of the study, inclusion criteria, and sample size. For these reasons, no review or meta-analysis has assessed the neural mechanisms underlying BP using functional neuroimaging. Therefore, this paper aims to fill this knowledge gap by reviewing studies using functional neuroimaging techniques to evaluate the neural substrates of the brain, explore the functional reorganization in BP patients, and help elucidate the neural mechanism of BP.

## Acknowledgements

We acknowledge the support and help of PROSPERO Review group and would like to appreciate the peer referees who provide review comments to improve the protocol.

## Author contributions

DH Lee and Joo-Hee Kim conceptualized the study and drafted the manuscript. BI Kwon produced figures for the manuscript. Yoo and Park developed the search strategy. JH Kim supervised the study and revised the manuscript accordingly. All authors have approved the final version of the manuscript.

**Conceptualization:** Dong Hyuk Lee, Joo-Hee Kim.

**Investigation:** Bo-In Kwon, Jun-Sang Yu, Sang Kyun Park.

**Supervision:** Joo-Hee Kim.

**Visualization:** Bo-In Kwon.

**Writing – original draft:** Dong Hyuk Lee.

**Writing – review & editing:** Dong Hyuk Lee, Joo-Hee Kim.
